# Comparative Plastomics of Ashwagandha (*Withania*, Solanaceae) and Identification of Mutational Hotspots for Barcoding Medicinal Plants

**DOI:** 10.3390/plants9060752

**Published:** 2020-06-15

**Authors:** Furrukh Mehmood, Abdullah, Zartasha Ubaid, Yiming Bao, Peter Poczai, Bushra Mirza

**Affiliations:** 1 Department of Biochemistry, Quaid-i-Azam University, Islamabad 45320, Pakistan; mfarrukh@bs.qau.edu.pk (F.M.); abd.ullah@bs.qau.edu.pk (A.); zartashaubaid.zu@gmail.com (Z.U.); 2 Botany Unit, Finnish Museum of Natural History, University of Helsinki, P.O. Box 7, FI-00014 Helsinki, Finland; 3 National Genomics Data Center, Beijing Institute of Genomics, Chinese Academy of Sciences, and China National Center for Bioinformation, Beijing 100101, China; baoym@big.ac.cn; 4 School of Future Technology, University of Chinese Academy of Sciences, Beijing 100049, China; 5 Vice Chancellor of Lahore College for Women University, Lahore 54000, Pakistan

**Keywords:** ashwagandha, chloroplast genome, indels, medicinal plants, mutational hotspots, phylogenomics, Solanaceae, substitutions, *Withania*

## Abstract

Within the family Solanaceae, *Withania* is a small genus belonging to the Solanoideae subfamily. Here, we report the de novo assembled chloroplast genome sequences of *W. coagulans, W. adpressa*, and *W. riebeckii.* The length of these genomes ranged from 154,162 to 154,364 base pairs (bp). These genomes contained a pair of inverted repeats (IRa and IRb) ranging from 25,029 to 25,071 bp that were separated by a large single-copy (LSC) region of 85,635–85,765 bp and a small single-copy (SSC) region of 18,457–18,469 bp. We analyzed the structural organization, gene content and order, guanine-cytosine content, codon usage, RNA-editing sites, microsatellites, oligonucleotide and tandem repeats, and substitutions of *Withania* plastomes, which revealed high similarities among the species. Comparative analysis among the *Withania* species also highlighted 10 divergent hotspots that could potentially be used for molecular marker development, phylogenetic analysis, and species identification. Furthermore, our analyses showed that even three mutational hotspots (*rps*4-*trn*T, *trn*M-*atp*E, and *rps*15) were sufficient to discriminate the *Withania* species included in current study.

## 1. Introduction

The globally distributed megadiverse Solanaceae family includes 93 genera and 2700 species [1,2,3]. The genus *Withania* Pauq., belonging to the subfamily Solanoideae, contains 10–20 species [1]. Among the worldwide list of *Withania* species, ashwagandha or winter cherry (*W. somnifera* (L.) Dunal) and paneer booti or ashutosh booti (*W. coagulans* (Stocks) Dunal) are considered highly important due to their therapeutic potential. *Withania* species are pivotal in the Ayurvedic medicine system in Southeast Asia, and *W. somnifera* has been used for medicinal purposes for around 3000 years [4,5]. Many studies of *Withania* have described various pharmacological properties of these species, (e.g., anti-inflammatory, anticancer, antidepressant, neuroprotective, and hepatoprotective) [6,7,8,9]. The ubiquity of such herbal products has expanded globally in recent decades. The worldwide market for medicinal plants is anticipated to reach 5 trillion USD by 2050, with Europe driving the market [10]. Although medicinal plants are outstanding sources of innovative drug development, assessing their pharmacological properties and effectiveness requires comprehensive approaches. Ashwagandha (*Withania* species) products show irregularities in quality through the herbal medicinal value chain affecting their impact and safety [11]. The quality of these manufactured herbal products is globally highly variable, and consistent analytical approaches are required to identify and monitor their quality along the value chain. The herbal medicine industry has considered DNA barcoding as a method that can be consistently applied in quality control over the manufactured products and to identify medicinal materials to protect consumers from dishonest suppliers. In addition, this method can also be used to identify toxic herbal materials in life-threatening situations, prevent poisoning, and improve control procedures of herbal drug substances [12].

The structure and composition of the chloroplast genome (or plastome) can be utilized to generate molecular markers that can be used in DNA barcoding [13]. Chloroplasts are important and universal organelles that are essential for photosynthesis. Chloroplasts are also associated with the synthesis of vitamins, pigments, fatty acids, and amino acids through various biochemical pathways [14]. Among many plant species, plastomes are 75–250 kilobases (kb) in size [15] and contain 120 genes, which include protein-encoding, ribosomal RNA (rRNA), and transfer RNA (tRNA) genes [16]. The structure of angiosperm plastomes varies from circular to linear even within the cells of the same individual [17]. Circular-formed plastomes show a typically quadripartite in structure, with two inverted repeats (IRs) separating the large single-copy (LSC) and a small single-copy (SSC) regions [15,18,19]. Plastomes show frequent variation in the number of tandem repeats, insertions and deletions (indels), single nucleotide polymorphism (SNPs), and as well as other rearrangements including translocations and inversion [19,20,21]. Plastomes have been termed as “super barcodes”, due to their comparatively conserved organization, gene content, adequate level of nucleotide substitution in protein-encoding genes, and uniparental inheritance, which make them excellent sources of phylogenetic reconstruction and species identification at diverse taxonomic levels [22,23,24,25]. Plastome-sequencing data can also be useful for agricultural trait improvement [26], transplastomics [27,28], population genetics [29], and conservation of species facing extinction [30].

Here, we aimed to assemble and compare the complete chloroplast genome sequences of *W. coagulans, W. adpressa* Coss., and *W. riebeckii* Schweinf. ex Balf.f. in addition to the previously reported *W. somnifera* genome [24]. We also sought to analyze the phylogenetic relationship of the genus among the Solanaceae family and to analyze differences in the organization of *Withania* plastomes such as repeats, indels, and substitutions, and to identify mutational hotspots for future DNA barcoding.

## 2. Materials and Methods

### 2.1. Genome Assembly and Annotation

Fresh green leaves of *W. coagulans* were obtained from Mianwali, Pakistan (32.5839° N 71.5370° E). The leaf segments were washed in 70% ethanol and total genomic DNA extraction was carried out according to the CTAB (cetyltrimethylammonium bromide) method of Lu et al. [31]. DNA quality and concentration were assessed by Colibri spectrometer Nanodrop (Titertek-Berthold, Berthold Detection Systems GmbH, Pforzheim, Germany) and 1% agarose gel electrophoresis. Genome sequencing was carried out by the Beijing Institute of Genomics using the Illumina HiSeq PE150 platform (Illumina Inc., San Diego, CA, USA). Furthermore, the Illumina sequence data of *W. adpressa* (5 Gb) and *W. riebeckii* (5 Gb) were acquired from the sequence read archive (SRA) deposited under accession numbers SRR8718119 and SRR8718120. The raw sequencing read quality was verified with the FastQC tool [32]. We used Velvet 1.2.10 [33] with k-mer sizes of 31, 41, 51, and 71 to initially assemble the large sequence contigs from raw paired-end reads. These contigs were then combined to produce complete chloroplast genomes though de novo assembly carried out with Geneious R8.1 (Biomatters Ltd., Auckland, New Zealand) [34]. The junction sites between LSC, SSC, and IR were determined for these novel assembled plastomes. Annotations of these genomes were performed, using GeSeq [35] and CPGAVAS2 [36]. The results were compared, inspected checking the start/stop codons manually. Additionally, tRNA genes were identified using tRNAscan-SE version 2.0 under default parameters [37] and ARAGORN version 1.2.38 [38]. CPGAVAS2 [36] and Clico FS [39] were used to draw circular maps of the genomes. The average coverage depths of the *Withania* species plastomes were determined by mapping the reads to the de novo assembled plastome through the Burrows–Wheeler aligner (BWA) [40] and visualizing in the Tablet [41]. The novel annotated plastomes were deposited in NCBI under the following accession numbers: *W. coagulans* (MN216390), *W. adpressa* (BK010847), and *W. riebeckii* (BK010849). The plastome of *W. coagulans* was also deposited in the GWH database of the National Genomics Data Center [42] (accession number GWHACBF00000000).

### 2.2. Comparative Chloroplast Genome Analysis

All de novo chloroplast genomes were aligned with multiple alignment using fast Fourier transform (MAFFT) 7.309 [43], using default parameters. Protein-encoding genes, intergenic spacer (IGS) regions, and introns were extracted to calculate the average number of nucleotide differences per site or nucleotide diversity (π) with a 100 bp window size as implemented in DnaSP v6 [44]. The substitution, transition (Ts), and transversion (Tv) rates were resolved from the MAFFT alignment, using *W. somnifera* as a reference. Each structural element, including the LSC, SSC, and IR, was aligned individually to analyze SNPs and indel polymorphisms with Geneious and DnaSP, respectively. The junction sites of the IRs and their border positions were compared using all *Withania* species and six additional Solanaceae outgroup species (Table S9), using the default setting of the IRscope [45]. The intergeneric comparison was carried out to gain insight to differences and syntenies that may exist between *Withania* and other Solanaceae species. Circoletto [46] was used to compare structural features of *Withania* chloroplast genomes using blastn search (e-value of <1 × 10^−10^) to create a Circos output.

The predictive RNA editor for plants-chloroplast genes (PREP-cp) was used to predict putative RNA editing sites using default settings [47], while codon usage and amino-acid frequencies were analyzed in Geneious R8.1. The ratios of synonymous (Ks) and non-synonymous (Ka) substitutions for each extracted protein-encoding gene were calculated with DnaSP for all *Withania*, using *W. somnifera* as reference. The data were interpreted as: Ka/Ks > 1, Ka/Ks = 1, Ka/Ks < 1, representing positive, neutral, and purifying selection, respectively. Microsatellites in *Withania* plastomes were detected with the microsatellite-web (MISA) [48], using a minimal repeat number of 7 for mononucleotide simple sequence repeats (SSRs), 4 for dinucleotide SSRs, and 3 for tri-, tetra-, penta-, and hexanucleotide SSRs. REPuter [49] was also used to locate forward (F), reverse (R), palindromic (P), and complementary (C) repeats with the following parameters: min. repeat size 30 bp, Hamming distance 3, min. similarity percentage of two repeat copies 90%, and max. computed repeats 500. A subsequent search for repeats was also carried out with tandem repeat finder [50] using default parameters.

### 2.3. Phylogenomic Analysis

We included all available *Withania* plastome sequences in our analysis and added further Solanaceae plastomes (Organelle Genome Resources of NCBI, accessed on 21 January 2020) from closely related groups of Physaleae and additional taxonomic groups from the so-called ‘x = 12 clade’. This group encompasses species of the traditional subfamily Solanoideae, *Nicotiana* L. and the Australian endemic tribe Anthocercideae belonging to Nicotianoideae. This strongly supported group is united with the cytological synapomorphy of chromosome numbers based on 12 pairs [1]. We used *Petunia* × *atkinsiana* (Sweet) D. Don ex W.H. Baxter (Syn.: *Petunia* × *hybrida* Vilm.) as an outgroup to root our tree, since this was the only available complete chloroplast genome sequence outside the x = 12 clade. For phylogenetic analysis, we removed one of the IR regions (IRa), and subsequently excised all protein-encoding genes from the plastomes. The reading frames were manually verified during extraction by checking the start and stop codons. We discarded *acc*D, *ycf*1, and *ycf*15 from our final alignment, because these genes were highly variable in size. The trans-spliced *rps*12 was also not included in the phylogenetic alignment together with sequence of the *inf*A pseudogene. The nucleotide sequences of 74 protein-coding genes were aligned with MAFFT (default setting) via the Geneious shell. IQ-TREE [51] was used to determine the best-fitting models for each partition of concatenated matrix using the TESTMERGEONLY and AICc (Akaike information criterion corrected for small sample sizes) options in the built-in ModelFinder [52]. The maximum likelihood (ML) tree search was carried out using the ultrafast bootstrap approximation (UFBoot; [53]) with 1000 replicates. UFBoot reduces computing time and provides an efficient alternative to standard bootstrap [53]. Branch supports were also assessed using the SH-like approximate likelihood ratio test (SH-aLRT), while final phylogenetic trees were edited using TreeDyn [54,55].

For further analyses we divided the dataset into protein-coding gene subsets according to the heuristic searches carried out with Partition Finder v1.1.1 [56] and default settings using Bayesian information criterion (BIC). Intron regions were regarded as distinct subsets. Partitioned Bayesian phylogenetic analyses were carried out with MrBayes v.3.2.3 [57]. jModelTest [58] was used with default settings to infer fitting substitution models (see Table S10). One cold and three heated Markov chains were run parallel with 2 × 10^6^ generations sampling every 100th tree per generation, with unlinked parameters across partitions. Branch length and topology parameters were set unlinked. Convergence was checked using the average standard deviation of split frequencies (ASDFs; <0.01) was used to measure convergence of the runs. A majority-rule consensus tree was constructed from the runs with a 25% burn-in removal.

## 3. Results

### 3.1. Organization and Characteristics of Withania Plastomes

**Figure 1:**
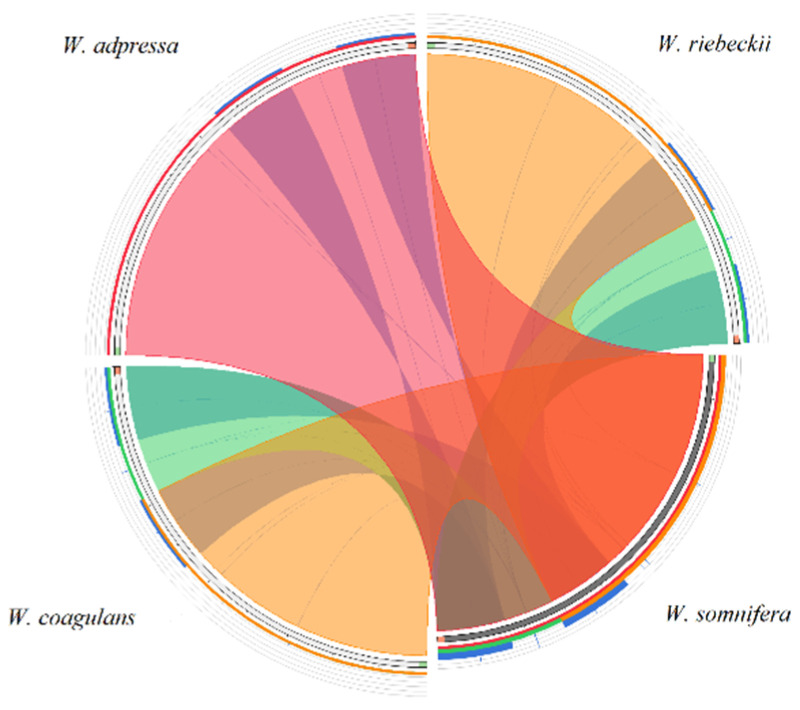
Structural comparison of *Withania* plastomes showing a high level of synteny and the lack of large rearrangements. The start and end points of the sequences are marked by green and orange blocks. The colored blocks outside the sequences refer to the score/max bit core ration, with green ≤ 0.50, orange ≤ 0.75, and red > 0.75. Blue blocks and chords represent the inverted repeats (IRs).

Our comparative analysis revealed that *Withania* species have similar plastome structures (Figure 1 and Table 1). The length of the assembled plastome varied between 154,162 and 154,364 bp. The average coverage depth of the assembled plastomes of *W. coagulans, W. adpressa*, and *W. riebeckii* was 573×, 566×, and 590×, respectively. The total guanosine-cytosine (GC) content of the de novo assembled *Withania* plastomes was 37.7%, as was the previously sequenced species.

**Figure 2:**
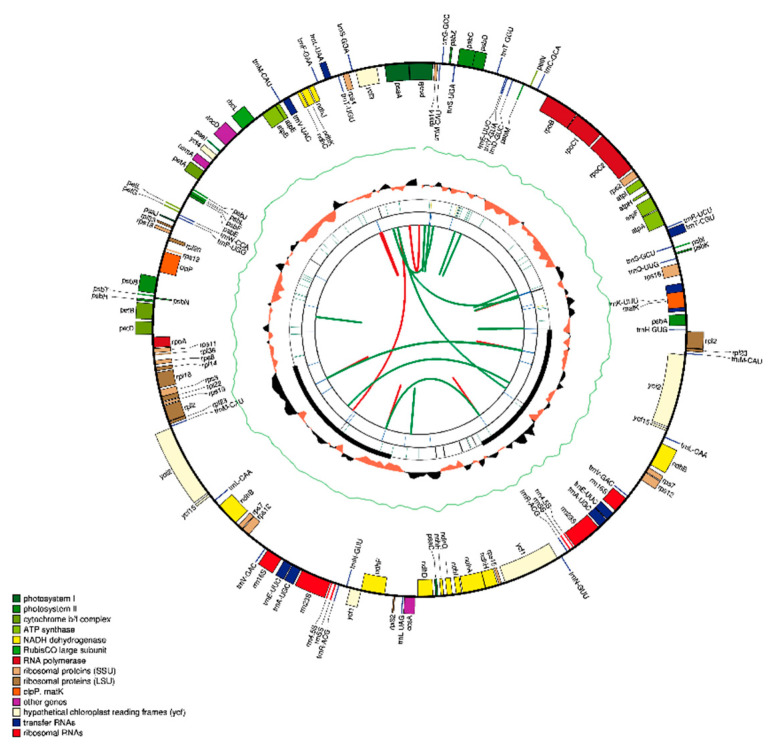
Consensus circular genome map of *Withania* plastomes. The first larger ring represents the gene structure of the chloroplast genome. The GC content is plotted as a green line while a skewed GC plot is presented as an orange/black track around the inner circles. The second ring is displaying the IRs as longer black boxes, while tandem repeats are represented by short bars. The third, and last circle shows the output of MISA microsatellite detection, where reverse repeats are connected with green arcs, while red arcs represent forward repeats.

**Figure 3:**
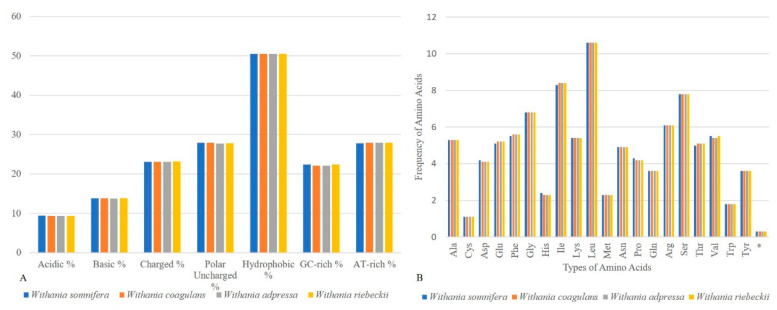
(**A**) Assessment of amino-acid groups and (**B**) amino-acid frequency comparison among *Withania* species.

The IRs showed a higher GC content (43.2%) compared to the large- (35.7%) and small-single copy regions (31.8%), which could have been due to the occurrence of rRNA genes containing GC-rich regions [19,59,60,61]. The plastomes of the de novo assembled *Withania* species had 132 genes from which 18 were represented in duplication in the inverted repeats (Table 2, Figure 2). All *Withania* plastomes contained 86 protein-encoding, 37 tRNA, and 8 rRNA genes. The IR regions contained 18 duplicated genes and out of these 7 were protein-encoding, 4 were rRNA, and 7 were tRNA genes. The *clp*P and *ycf*3 genes had two introns in their nucleotide sequence, while *rps*16, *atp*F, *rpo*C1, *pet*B, *pet*D, *rpl*16, *ndh*A, *rpl*2, and *ndh*B had only one. The 5′ end exon of the trans-spliced gene *rps*12 was found in the LSC and the 3′ end exons were located in the IR. The GC content was the highest among tRNAs (53%) and rRNAs (55.3%). Hydrophobic amino acids were abundant, while the acidic amino acids were present in the least amount in plastomes of the genus *Withania*. These amino acids were adenine-thymine (AT)-rich sequences in all species (Figure 3A). The analysis of codon usage and amino acids frequencies indicated leucine (Leu) as the most frequent and cysteine (Cys) as a rare amino acid in *Withania* plastomes (Figure 3B). The codon usage also revealed a shift towards A/T at the third codon position (Table S1).

### 3.2. Divergence Hotspots in Withania

Our comparison showed that all *Withania* genomes had similar nucleotide compositions in all structural (LSC, SSC, and IR) and coding regions, which extended even to IGSs (Table S2). The number of substitutions ranged between 25 and 116, while substitution types were shared among species (Table 3). A/G and C/T SNPs occurred frequently among the genomes (Table 3), while the ratio of Ts and Tv in the plastomes ranged from 1.04 to 1.25 in the LSC and between 0.5 and 1.5 in the SSC; the ratio varied from 1.3 to 2 in the IR region (Table S3). In general, Ts were more frequent in *Withania*, consistent with observations in other plant species [61,62]. Indels were frequent in the LSC region and their number ranged from 32 to 46. The IRs contained only a few indels (Table 4). This may have been due to the observation that IR sequences evolve under concerted evolution compared to LSC and SSC regions that contain more substitutions [63]. When all positions with single- or multinucleotide variations as SNPs were considered, 207 SNPs were identified, which corresponds to a mean SNP frequency of 0.2070 SNPs/kb in *Withania* species. Indels showed a mean frequency of 0.116/kb.

**Figure 4:**
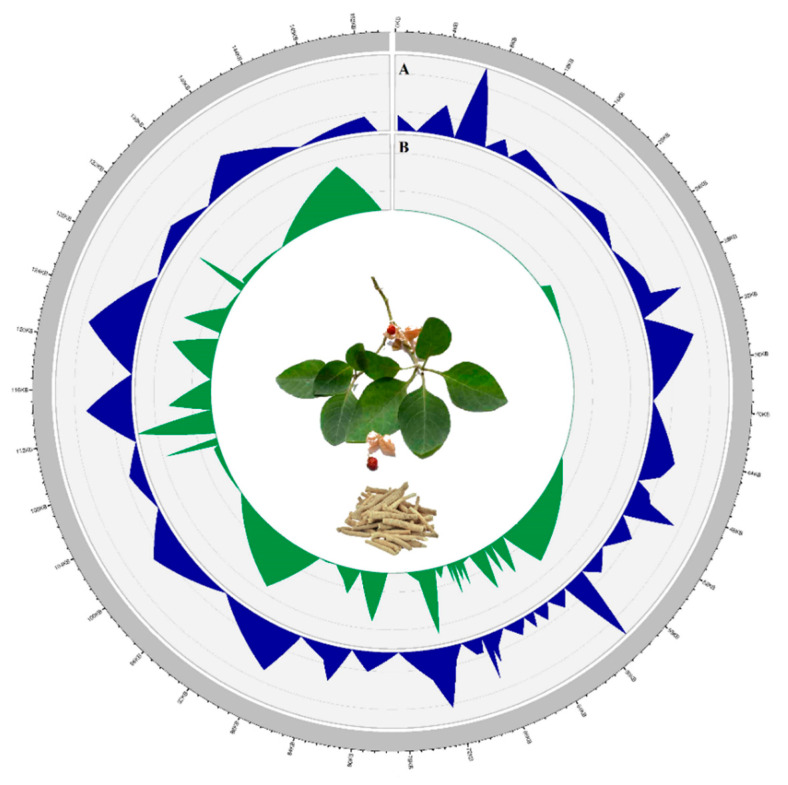
Circular visualization of the distribution of polymorphisms among *Withania* species. The plastome is shown as a size-proportioned (bp) grey bar in the outer circle. In the inner circle, the values of the (**A**) average number of nucleotide differences per site or nucleotide diversity (π) and (**B**) indel diversity are shown with a 100 bp window size.

The indels and SNP mutational events in the plastome showed uneven distributions and clustered as “hotspots” [64,65]. These fast evolving regions are ideally suited for DNA barcoding [66]. The IGS were more polymorphic (average π = 0.0027) than protein-coding regions (π = 0.0011) and introns (average π = 0.0015). Among the *Withania* species, the values ranged from 0.0003 (*psa*B) to 0.0119 (*ndh*l-*ndh*A region; Figure 4). We selected the 10 most polymorphic regions for further investigation based on the analysis of mutation rates of the complete chloroplast genome sequences (Table 5). From the selected regions, nine were IGSs, and one was a protein-coding gene (*rps*15). We assessed the efficacy of these regions to discriminate among the four species of *Withania* and found that three regions (*rps*4-*trn*T, *trn*M-*atp*E, and *rps*15) provided enough information for successful barcoding.

We also investigated the Ks and Ka substitutions and their ratio (Ka/Ks; Table S4). We selected 77 protein-encoding genes for further analysis and observed that 69 genes had Ks = 0 and 58 had Ka = 0, while 72 genes had both Ks and Ka = 0. Of the protein-encoding genes, four (*acc*D, *ycf*2, *ycf*1, and *ndh*F) had Ka/Ks ratios > 1. *ycf*1 and *psb*C showed Ka/Ks ratios > 0–1 for *W. riebeckii, ndh*F for *W. coagulans*, and *rps*15 for *W. adpressa* and *acc*D, *ycf*2 showed Ka/Ks > 0 for *W. adpressa* and *W. riebeckii,* and *rpo*C2 for *W. coagulans* and *W. riebeckii*. The low divergence of most chloroplast genes showed signs of purifying selection to conserve the sequence and function of proteins related to photosynthesis.

### 3.3. Repeat Structure and Analyses

**Figure 5:**
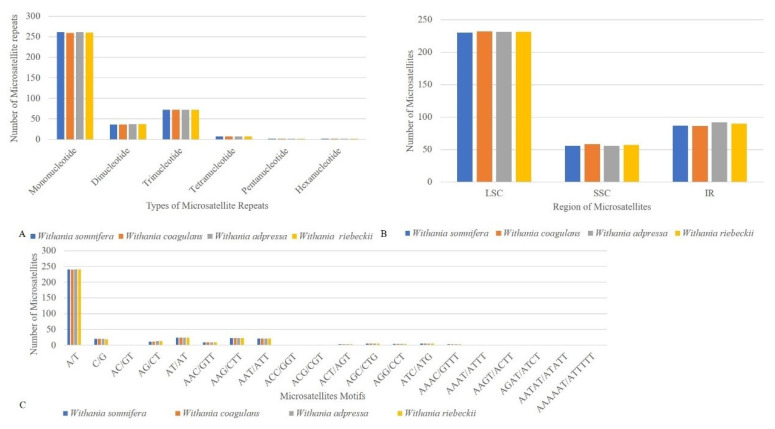
Comparative analysis of microsatellite repeats among *Withania* species. (**A**) Total number of microsatellites and their classification according to the number of repeat units. (**B**) The distribution of microsatellites among structural regions of the plastome. (**C**) Repeat unit composition of *Withania* microsatellites.

**Figure 6:**
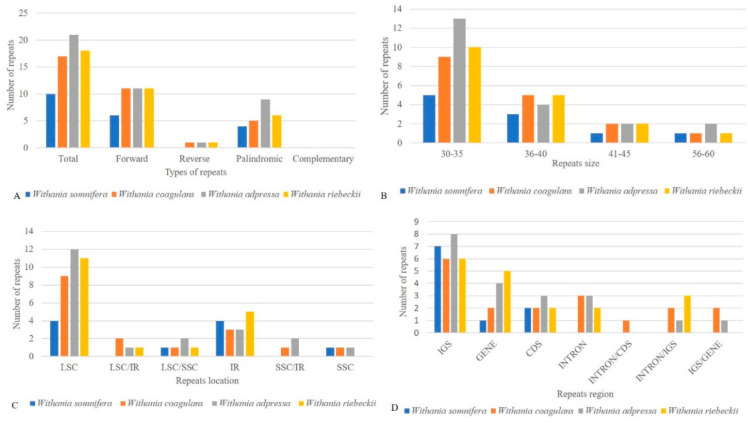
(**A**) The total number of oligonucleotide repeats among *Withania* species and their distribution according to specific characteristics. (**B**) The distribution of repeats in size ranges. (**C**) The number of repeats grouped according to their location in each structural region (**D**) The distribution of repeats in intergenic spacer regions (IGS), genes, coding DNA sequences (CDS), and introns and their proportionate occurrence.

**Figure 7:**
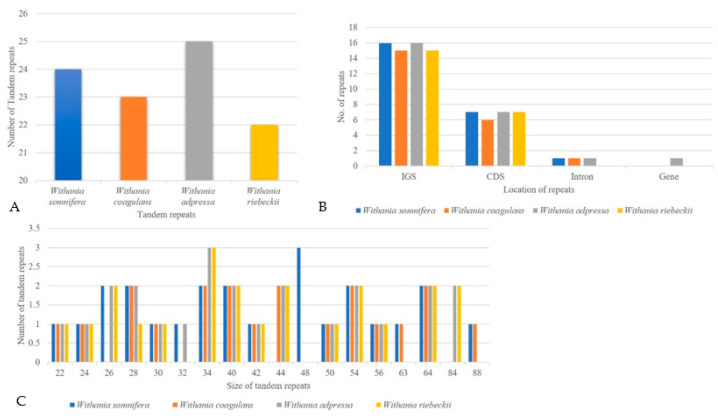
The assessment of tandem repeats (**A**) The total number of tandem repeats and (**B**) their distribution among functional regions of the plastome. (**C**) Tandem repeat number, size, and distribution.

Chloroplast repeat sequences are important sources of variation for evolutionary studies, plant breeding, and construction of linkage maps [67,68,69]. We performed a microsatellite analysis that revealed shared microsatellite loci ranging from 376 (*W. coagulans*) to 379 (*W. adpressa*). Poly-A and T SSR motifs were frequent in *Withania* chloroplast genome sequences, while AT/TA dinucleotide stretches were also highly abundant. The mononucleotide motifs occurred in 7–17-unit repeats, while dinucleotide repeats had a frequent 4–5-units, whereas other types of SSRs were present mainly in 3–5-unit repeats. Most SSRs occurred in the LSC, followed by IR and SSC (Figure 5) (Table S5). REPuter was also employed to locate further tandem repeats in all *Withania* species. A total of 66 oligonucleotide repeats were found among *Withania* species. The F and P repeats were present in large numbers in all species. The oligonucleotide repeats were variable in size (30–60 bp) and a large fraction of the repeats was located in the LSC and existed in IGS regions, followed by gene, intron, and coding DNA sequence (CDS) regions (Figure 6; Table S7). The number of tandem repeats varied from 22 to 25 bp among *Withania* species (Figure 7; Table S6).

### 3.4. Comparative Plastomics and Inverted Repeat Boundaries

**Figure 8:**
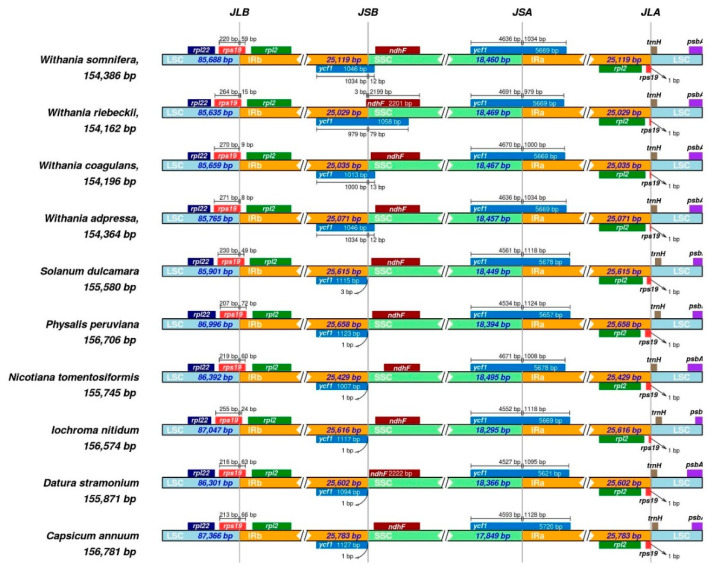
The comparative assessment of junction sites among six *Withania* species and six outgroups of Solanaceae. Genes are represented by boxes above (negative-strand) and below (positive-strand) the proportionate line. The size of each gene and their relative position at the junctions are shown in base pairs (bp). The figure shows the conservation of the junctions among *Withania* species with mild shifts at the JSB (IRb/SSC).

The plastome of land plants has a conserved quadripartite structure while variation frequently occurs in a form of expansion and contraction of the junction sites that could rarely even lead to the loss of the entire IR regions [70,71]. The size of each structural element of the plastome (LSC, SSC, and IR) shows variation at the junction sites JL (LSC/IR) and JS (IR/SSC). Studying these boundaries among plant linages could broaden our knowledge about chloroplast genome evolution and speciation processes [72]. Syntenies among the junction sites could be conserved between species and could explain relationships among them [73]. To investigate such events, we compared the JL (LSC/IR) and JS (IR/SSC) junction sites of *Withania* plastomes (Figure 8). The resemblance at junctions revealed the close resemblance among the *Withania* species. The *rps*19 gene was found at the junction site of JLB (LSC/IRb), and a portion of this gene (8–59 bp) was copied in the IRa in all *Withania* genomes. The *ndh*F gene was entirely present in the SSC region in *W. somnifera* and *W. adpressa*, but in *W. coagulans* (5 bp) and *W. riebeckii* (3 bp) it was located in the IRb region.

### 3.5. Putative RNA-Editing Sites

RNA editing is the molecular processes that can alter the sequence of the transcribed RNA by insertion, deletion, or nucleotide substitution [46]. RNA editing aids in creating transcripts and maintaining protein diversity [74], thus several sites are conserved in the plastome of angiosperms [75]. To examine the RNA editing in *Withania* species, we predicted putative sites in the plastomes using PREP-cp. This revealed 37 putative sites in 15 genes of *W. somnifera,* while 35, 39, and 37 editing sites were found in 13 genes of *W. coagulans* and in 14 genes of *W. adpressa* and *W. riebeckii*, respectively. The gene *clp*P has editing sites only in *W. somnifera* and *ccs*A only in *W. adpressa.* Among *Withania* species *ndh*B (9), *ndh*D (7), and *rpo*B (5) had the highest number of RNA-editing sites. All species had high levels of conversion for serine (Ser) to leucine (Leu; 60%, 53.8%, and 59.4%, respectively), followed by proline (Pro) to Leu (14.28%, 17.94%, and 16.21%, respectively), and Ser to phenylalanine (Phe; 8.57%, 10.2%, and 10.8%, respectively). Of the putative RNA-editing sites detected, 33 (94.2%), 34 (87.1%), and 33 (89.1%) codons were substituted on the second nucleotide and two (5.71%), five (12.8%), and four (10.81%) codons were substituted in the first nucleotide in *W. coagulans, W. adpressa*, and *W. riebeckii*, respectively. Many amino acids were converted from Ser to Leu helping to form hydrophobic amino acids, e.g., valine (Val), Leu, and Phe (Table S8).

### 3.6. Phylogenetic Analysis

**Figure 9:**
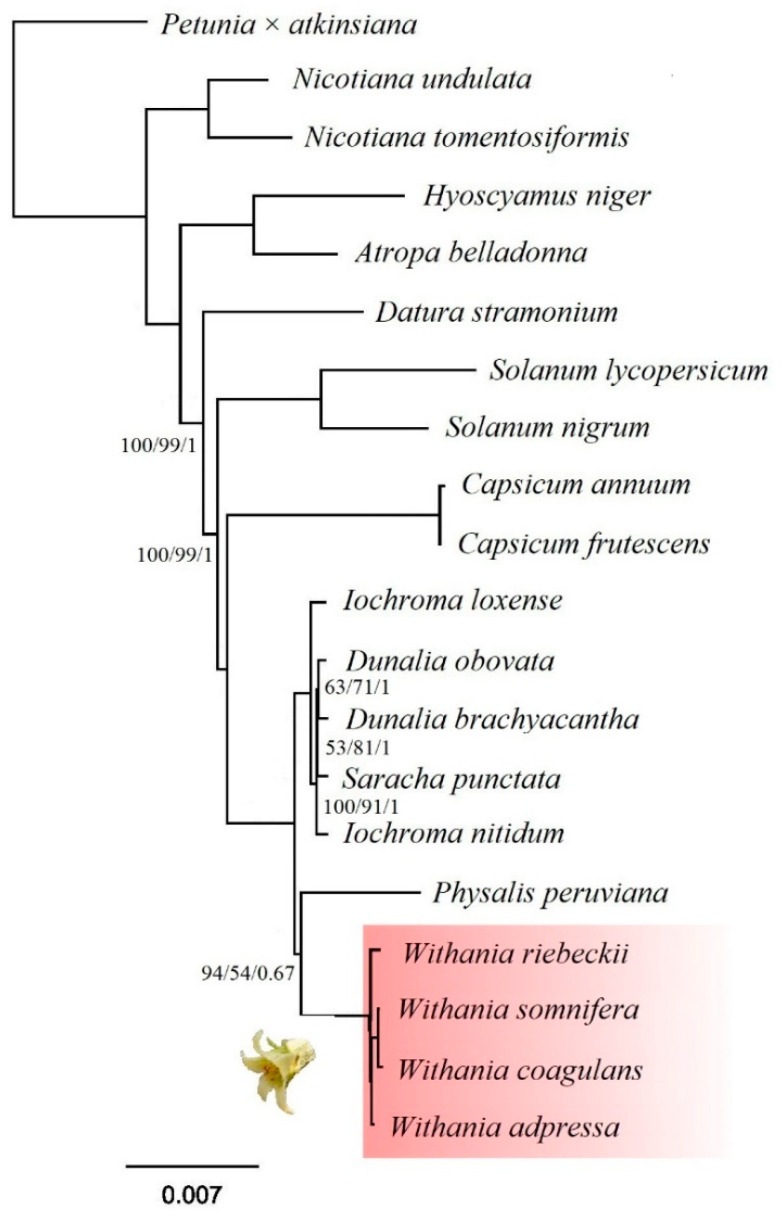
Maximum likelihood (ML) tree reconstructed among 19 species of Solanaceae based on 74 protein-coding genes. Bootstrap, SH-aLRT values and Bayesian posterior probabilities are indicated at each node as UFBoot/SH-aLRT/PP. Unmarked nodes have maximum support values. The scale bar is proportional to substitution per site.

We performed maximum-likelihood (ML) and Bayesian analysis for phylogenetic reconstruction for 19 Solanaceae species, based on selected protein-coding gene sequences extracted from whole-plastome sequences. Based on a 69,582-bp alignment, our tree was reconstructed and resolved identical topologies for both methods and a phylogenetic tree were supported by high bootstrap values and posterior probabilities (Figure 9). The genus *Withania* was represented by *W. adpressa*, native to North Africa, Morocco, and Algeria, *W. coagulans* from the eastern distribution area, *W. riebeckii* native to the island of Socotra, Jemen, and finally, the widespread *W. somnifera.* Our phylogenetic analysis with limited taxonomic sampling resolved *Withania* as a monophyletic of the genus. However, further sampling is needed to investigate the relationship of the allied genera especially from *Athenaea* Sendtn., *Aureliana* Sendtn., and *Mellissia* Hook. f. not included in our analysis, which were shown to be closely related to *Withania* [76]. Our results were consistent with previous findings based on plastid intergenic *atp*B-*rbc*L spacer [77], *ndh*F and *trn*LF [1], or whole plastome sequences [18].

## 4. Discussion

Chloroplast DNA (cpDNA) is frequently used in plant phylogenetics at various levels (i.e., generic level and above) [19,66,78]. It has also been used in Solanaceae systematics to infer family level phylogenetic relationships and to identify major clades and dispersal events [79]. Thus, we characterized, annotated, and analyzed the plastome of *Withania* species, which was further used in phylogenetic inference. *Withania* species belong to a rather diverse and widely distributed Withaninae clade within the so-called physaloid group. Species of the genus *Withania* are morphologically similar: the flowers are found in lateral clusters (fascicles) lacking a supporting inflorescence stem (peduncle). The flower petals (corollas) are bell or elongated cup-shaped, sometimes urn-shaped, circular and flattened (rotate), or trumpet-shaped (salverform), while the filaments often form nectar grooves with lateral attachments. The Withaninae clade consists of approximately seven small often monotypic genera mostly found in the Old World, e.g., *Tubocapsicum* (Wettst.) Makino, *Mellissia*, *Aureliana,* or *Discopodium* Hochst. D’Arcy [80] considered *Withania* to be one of the truly Old-World genera, while Symon [81] regarded it as a distinctive African Gondwanan element. *Withania* has a center of distribution around Spain, NW Africa extending to the Canary Islands, while another is located in India, the southern region of the Arabian Peninsula, and the Horn of Africa. The phylogenetic relationships within the genus are poorly known, and the biology, chromosome numbers, and the exact number of species are also lacking. Chromosome counts showed that most species of *Withania* are polyploids with 2n = 2x = 48 [82], derived from the x = 12 haploid chromosome number typical for the majority of Solanaceae species. In addition to the currently accepted *Withania* taxa, there are 35 unresolved botanical names that need further investigation to clarify the taxonomy of the genus.

In the Hepper’s treatment [83], *Withania* consisted of 10 species, which were extended by Hunziker [84] with the addition of nine mesophytes from the genera *Mellissia* Hook. and *Physaliastrum* Makino. These additions extended the geographical range of the genus from the Canary Islands in the west, through Asia to China and Japan in the east. Symon [81] also emphasized the similarity of *Mellissia* (a critically endangered endemic of St Helena) to *Withania* but retained them as distinct genera. In contrast, Hunziker [84] included *Mellissia* within *Withania* and molecular results support this placement [1]. There is no consensus on the positions of the small clades related to *Withania*, while its closest relatives are also debated. In our analysis, *Withania* formed a clade together with *Physalis,* similarly to the findings of Deanna et al. [82], although this branching is supported by only weak bootstrap values.

Plastome sequences could be used as tools to further elucidate species boundaries and investigate the phylogenetic relationships among the small clades of Withaninae and resolve the taxonomic debate over the placement of *Melissia* and other monotypic genera. For such barcoding studies our results could provide valuable reference genomes for assemblies. The hotspot regions described in our study could be useful in such phylogenetic or even population genetic investigations. It was previously demonstrated that identifying highly variable regions by comparative plastomics could provide reveal loci that could be used in DNA barcoding [85,86,87]. Such divergent hotspots in the plastomes can be applied for DNA barcoding at the generic level [29,88,89,90]. Thus, the set of 10 polymorphic regions identified among *Withania* in our study could be applied for DNA barcoding. Moreover, similar to the aforementioned studies, our identified mutational hotspots showed high discrimination properties and from the 10 mutational hotspots, three regions were found to be sufficient for the identification of the four *Withania* species included in our study.

We analyzed Ks substitutions and Ka substitutions of protein-coding genes and recorded greater Ks substitutions relative to the Ka substations. Such observations are essential markers in evolution for defining slow- and fast-evolving genes [91]. The Ka/Ks ratio also informs us about the selection pressure on these genes. When the Ka/Ks value is minimal, it represents purifying selection, while values similar to it or equal to 1 represent neutral evolution, and values greater than 1 denote positive selection [85]. Most plastid genes showed a minimal Ka/Ks ratio (<1), demonstrating that purifying selection is acting over these genes, due to functional constraints of the plastome. However, *atp*B, *ndh*D, *ndh*F, *rpo*A, *rpo*C1, *rps*2, and *rps*12 showed greater Ka/Ks values (>1), possibly indicating selective pressure acting over these genes that was previously proposed in other groups [92,93,94,95]. Our sampling in the *Withania* clade was limited to explore the biological causes of the elevated Ka/Ks ratios observed in cases involving these genes. Here, we suggest that the following set of genes could be the principal candidates in investigations of these environmental interactions and their effects on plastid genes. Such investigations should include a nearly complete phylogenetic sampling of *Withania* and consider the effects of arbitrary variations in Ka and Ks values leading to false positive inferences and values higher than 1. These shortcoming can be bypassed by complete sampling and additional tests of selective pressure also stressed here for future analyses.

## 5. Conclusions

It has been shown that DNA barcoding can fail in complicated groups [96]. Solanaceae includes many species complexes with tangled taxonomy such examples of species groups can be found in potatoes and its wild relatives, e.g., *Solanum brevicaule* complex [97], or the eggplant and its wild relatives (*S. melongena* complex [98]) but in other clades of the family for example in the genus *Petunia* [99] or closely related *Physalis* [100]. In these complicated groups well known plastid barcode regions (e.g., *trn*H-*psb*A, *mat*K) could lack enough polymorphism and thus could fail to provide species-specific information necessary for differentiation [96]. It has been shown that plastid genome based “super barcoding” could overcome these difficulties and could differentiate species in difficult taxonomic groups. This approach has been successfully employed in the *S. melongena* complex to trace the ancestors of cultivated eggplant and differentiate closely related wild species [98]. Here, we compared the complete plastome sequences of four *Withania* species and investigated if plastid genomes based “super barcoding” could be applied among closely related ashwagandha species. The structure of these genomes showed synteny with a previously reported organization of Solanaceae species. We identified sequence divergence hotspots and located repeat sequences and indels in the plastomes of *Withania* species. These regions may constitute a useful means to develop suitable molecular markers for species identification and DNA barcoding of ashwagandha medicinal products. It is hoped that our study will aid the development of DNA barcoding markers to clarify the taxonomic identity of *Withania* species in medicinal plant production. Such plastome-based “super barcoding” could be repeatable, reliable, and sensitive enough to distinguish look-alike species of ashwagandha.

## Figures and Tables

**Table 1:** Summary of *Withania* chloroplast genome features.

Characteristics		*W. somnifera*	*W. coagulans*	*W. adpressa*	*W. riebeckii*
Size (base pair; bp)		154,386	154,196	154,364	154,162
LSC length (bp)		85,688	85,659	85,765	85,635
SSC length (bp)		18,464	18,467	18,457	18,469
IR length (bp)		25,117	25,035	25,071	25,029
Number of genes		132	132	132	132
Protein-coding genes		86	86	86	86
tRNA genes		37	37	37	37
rRNA genes		8	8	8	8
Duplicate genes		18	18	18	18
GC content	Total (%)	37.7%	37.7%	37.7%	37.7%
	LSC (%)	35.7%	35.7%	35.7%	35.7%
	SSC (%)	31.8%	31.8%	31.8%	31.8%
	IR (%)	43.2%	43.2%	43.2%	43.2%
	CDS (%)	38.2%	38.2%	38.2%	38.2%
	rRNA (%)	55.3%	55.3%	55.3%	55.3%
	tRNA (%)	53%	52.9%	53%	53%
	All gene (%)	40%	39.8%	39.8%	39.8%
Protein coding part (CDS) (%bp)		50.9%	51.0%	51.0%	51.0%
All gene (%bp)		72.06%	72.11%	72.07%	72.13%
Non-coding region (%bp)		27.94%	27.89%	27.93%	27.87%

**Table 2:** Genes and their functional categories in *Withania* chloroplast genomes.

Category for Gene	Group of Gene	Name of Gene				
Photosynthesis-Related Genes	Photosystem I	*psa*A	*psa*B	*psa*C	*psa*I	*psa*J
	Photosystem II	*psb*A	*Psb*B	*psb*C	*psb*D	*psb*E
		*psb*F	*psb*H	*psb*I	*psb*J	*psb*K
		*psb*L	*psb*M	*psb*N		
	Cytochrome b/f complex	*psb*T	*psb*Z	*pet*N	*pet*A	*pet*L
		*pet*G	*pet*D *	*pet*B *		
	ATP synthase	*atp*I	*atp*H	*atp*A	*atp*F *	*atp*E
		*atp*B				
	Assembly/stability of photosystem I	*ycf*3 **	*ycf*4			
	NADPH dehydrogenase	*ndh*B *^,a^	*ndh*H	*ndh*A *	*ndh*I	*ndh*G
		*ndh*J	*ndh*E	*ndh*F	*ndh*C	*ndh*K
		*ndh*D				
	Rubisco	*rbc*L				
Transcription and Translation Related Genes RNA Genes	Transcription Small subunit of ribosome	*rpo*A	*rpo*C2	*rpo*C1 *	*rpo*B	*rps*16 *
		*rps*7 ^a^	*rps*15	*rps*19	*rps*3	*rps*8
		*rps*14	*rps*11	*rps*12 ^a,^*	*rps*18	*rps*4
		*rps*2				
	Large subunit of ribosome	*rpl*2 ^a,^*	*rpl*23 ^a^*^,^*	*rpl*32	*rpl*22	*rpl*14
		*rpl*33	*rpl*36	*rpl*20	*rpl*16 *	
	Ribosomal RNA	*rrn*16 ^a^	*rrn*4.5 ^a^	*rrn*5 ^a^	*rrn*23 ^a^	
	Transfer RNA	*trn*V-GAC ^a^	*trn*I-CAU *	*trn*A-UGC ^a,^*	*trn*N-GUU ^a^	*trn*P-UGG
		*trn*W-CCA	*trn*V-UAC *	*trn*L-UAA *	*trn*F-GAA	*trn*R-ACG ^a^
		*trn*T-UGU	*trn*G-UCC ^a,^*	*trn*T-GGU	*trn*R-UCU	*trn*E-UUC
		*trn*Y-GUA	*trn*D-GUC	*trn*C-GCA	*trn*S-GCU	*trn*H-GUG
		*trn*K-UUU	*trn*Q-UUG	*trnf*M-CAU	*trn*G-GCC	*trn*S-UGA
		*trn*S-GGA	*trn*F-GAA	*trn*M-CAU	*trn*L-CAA *	
		*trn*I-GAU *^,a^	*trn*L-UAG			
Other Genes	RNA processing	*mat*K				
	Carbon metabolism	*cem*A				
	Fatty acid synthesis	*acc*D				
	Proteolysis	*clp*P **				
	Component of TIC complex	*ycf*1 ^a^				
	Hypothetical proteins	*ycf*2 ^a^	*Ycf*15			

* Gene with one intron, ** gene with two introns, **^a^** gene with two copies, same genes in all *Withania* species.

**Table 3:** Substitution and their distribution in *Withania* plastomes, compared to the *W. somnifera* as reference.

Types	*W. coagulans*	*W. adpressa*	*W. riebeckii*
A/G	4	19	28
C/T	10	15	30
A/C	3	11	18
C/G	2	2	3
G/T	4	14	25
A/T	2	5	12
Total	25	66	116
**LSC**	20	43	79
**SSC**	5	20	30
**IR**	0	3	7

**Table 4:** Insertions and deletions among *Withania* species compared to the *W. somnifera* reference genome.

	***W. coagulans***	**Indel Length (bp)**	**Indel Average Length**
LSC	27	133	4.926
SSC	3	61	20.33
IR	2	82	41.00
	***W. adpressa***	**Indel Length (bp)**	**Indel Average Length**
LSC	27	143	5.296
SSC	6	11	1.833
IR	5	68	13.60
	***W. riebeckii***	**Indel Length (bp)**	**Indel Average Length**
LSC	34	213	5.917
SSC	6	103	17.16
IR	4	106	26.50

**Table 5:** Mutational hotspots among *Withania* species.

S. No	Region	Nucleotide Diversity	T. No’s of Mutation	Region Length
1	*ndh*I-*ndh*A	0.0119	2	84
2	*trn*Y-GUA-*trn*E-UUC	0.0085	1	59
3	*rpl*14-*rpl*16	0.0080	2	125
4	*rps*19-*rpl*2	0.0071	1	70
5	*rps*15	0.0064	3	261
6	*trn*M-CAU-*atp*E	0.0053	2	221
7	*rps*4-*trn*T-UGU	0.0051	3	364
8	*trn*Q-UUG-*psb*K	0.0048	3	346
9	*ndh*H-*rps*15	0.0045	1	111
10	*trn*G-GCC-*trn*R-UCU	0.0041	1	164

## Supplementary Materials

The following are available online at https://www.mdpi.com/2223-7747/9/6/752/s1, Table S1: Base composition in the chloroplast genome of *W. somnifera*, *W. coagulans*, *W. adpressa* and *W. riebeckii*; Table S2: Codon usage in the chloroplast genome of *W. somnifera*, *W. coagulans*, *W. adpressa* and *W. riebeckii*; Table S3: Putative RNA editing sites composition in the chloroplast genome of *W. somnifera*, *W. coagulans*, *W. adpressa* and *W. riebeckii*; Table S4: Comparison of synonymous and non-synonymous substitution in *Withania* species S5: Microsatellites loci in the chloroplast genome of *W. somnifera*, *W. coagulans*, *W. adpressa* and *W. riebeckii*; Table S6: Oligonucleotide repeats in chloroplast genomes of *W. somnifera*, *W. coagulans*, *W. adpressa* and *W. riebeckii*; Table S7: Tandem Repeat sequences in the chloroplast genome of *W. somnifera*, *W. coagulans*, *W. adpressa* and *W. riebeckii*; Table S8: The distribution and characteristics of SNPs found among *Withania* plastid genomes; Table S9: NCBI GenBank accession numbers used in this study; Table S10: Model selected for each data partition identified by software Partition Finder for protein-coding chloroplast genes and intron regions in maximum likelihood (ML) analysis and Bayesian inference (BI).

## References

[1] Olmstead R.G., Bohs L., Migid H.A., Santiago-Valentin E., Garcia V.F., Collier S.M. (2008). A molecular phylogeny of the *Solanaceae*. Taxon.

[2] Olmstead R.G., Bohs L. A summary of molecular systematic research in *Solanaceae*: 1982–2006. *Solanaceae* IV: Genomics meets biodiversity. Proceedings of the VIth International *Solanaceae* Conference.

[3] Knapp S., Bohs L., Nee M., Spooner D.M. (2004). *Solanaceae*—A model for linking genomics with biodiversity. Comp. Funct. Genom..

[4] Mirjalili M.H., Moyano E., Bonfill M., Cusido R.M., Palazón J. (2009). Steroidal lactones from *Withania somnifera*, an ancient plant for novel medicine. Molecules.

[5] Singh P., Guleri R., Singh V., Kaur G., Kataria H., Singh B., Kaur G., Kaul S.C., Wadhwa R., Pati P.K. (2015). Biotechnological interventions in *Withania somnifera* (L.) dunal. Biotechnol. Genet. Eng. Rev..

[6] Uddin Q., Samiulla L., Singh V.K., Jamil S.S. (2012). Phytochemical and pharmacological profile of *Withania somnifera* dunal: A review. J. Appl. Pharm. Sci..

[7] Maurya R. (2010). Chemistry and pharmacology of *Withania coagulans*: An ayurvedic remedy. J. Pharm. Pharm..

[8] Ul-Haq I., Youn U.J., Chai X., Park E.-J., Kondratyuk T.P., Simmons C.J., Borris R.P., Mirza B., Pezzuto J.M., Chang L.C. (2013). Biologically active withanolides from *Withania coagulans*. J. Nat. Prod..

[9] Rehman S., Keefover-Ring K., Haq I.U.L., Dilshad E., Khan M.I., Akhtar N., Mirza B. (2019). Drier climatic conditions increase withanolide content of *Withania coagulans* enhancing its inhibitory potential against human prostate cancer cells. Appl. Biochem. Biotechnol..

[10] Shinwari Z.K. (2010). Medicinal plants research in Pakistan. J. Med. Plants Res..

[11] Raclariu A.C., Heinrich M., Ichim M.C., de Boer H. (2018). Benefits and limitations of DNA barcoding and metabarcoding in herbal product authentication. Phytochem. Anal..

[12] Li M., Cao H., But P.P.H., Shaw P.C. (2011). Identification of herbal medicinal materials using DNA barcodes. J. Syst. Evol..

[13] Jin S., Daniell H. (2015). The engineered chloroplast genome just got smarter. Trends Plant. Sci..

[14] Cooper G. (2000). Chloroplasts and Other Plastids in the Cell: A Molecular Approach.

[15] Palmer J.D. (1985). Comparative organization of chloroplast genomes. Annu. Rev. Genet..

[16] Daniell H., Lin C.-S., Yu M., Chang W.-J. (2016). Chloroplast genomes: Diversity, evolution, and applications in genetic engineering. Genome Biol..

[17] Oldenburg D.J., Bendich A.J. (2015). DNA maintenance in plastids and mitochondria of plants. Front. Plant. Sci..

[18] Amiryousefi A., Hyvönen J., Poczai P. (2018). The chloroplast genome sequence of bittersweet (*Solanum dulcamara*): Plastid genome structure evolution in Solanaceae. PLoS ONE.

[19] Abdullah, Shahzadi I., Mehmood F., Ali Z., Malik M.S., Waseem S., Mirza B., Ahmed I., Waheed M.T. (2019). Comparative analyses of chloroplast genomes among three *Firmiana* species: Identification of mutational hotspots and phylogenetic relationship with other species of *Malvaceae*. Plant. Gene.

[20] Henriquez C.L., Abdullah, Ahmed I., Carlsen M.M., Zuluaga A., Croat T.B., Mckain M.R. (2020). Evolutionary dynamics of chloroplast genomes in subfamily Aroideae (Araceae). Genomics.

[21] Xu J.-H., Liu Q., Hu W., Wang T., Xue Q., Messing J. (2015). Dynamics of chloroplast genomes in green plants. Genomics.

[22] Moore M.J., Bell C.D., Soltis P.S., Soltis D.E. (2007). Using plastid genome-scale data to resolve enigmatic relationships among basal angiosperms. Proc. Natl. Acad. Sci. USA.

[23] Ravi V., Khurana J.P., Tyagi A.K., Khurana P. (2008). An update on chloroplast genomes. Plant. Syst. Evol..

[24] Mehmood F., Abdullah, Shahzadi I., Ahmed I., Waheed M.T., Mirza B. (2020). Characterization of *Withania somnifera* chloroplast genome and its comparison with other selected species of *Solanaceae*. Genomics.

[25] Iram S., Hayat M.Q., Tahir M., Gul A., Abdullah, Ahmed I. (2019). Chloroplast genome sequence of *Artemisia scoparia*: Comparative analyses and screening of mutational hotspots. Plants.

[26] Bansal K.C., Saha D. (2012). Chloroplast genomics and genetic engineering for crop improvement. Agric. Res..

[27] Waheed M.T., Thönes N., Müller M., Hassan S.W., Razavi N.M., Lössl E., Kaul H.P., Lössl A.G. (2011). Transplastomic expression of a modified human papillomavirus L1 protein leading to the assembly of capsomeres in tobacco: A step towards cost-effective second-generation vaccines. Transgenic Res..

[28] Waheed M.T., Ismail H., Gottschamel J., Mirza B., Lössl A.G. (2015). Plastids: The green frontiers for vaccine production. Front. Plant. Sci..

[29] Ahmad I. (2014). Evolutionary Dynamics in Taro. Ph.D. Thesis.

[30] Wambugu P.W., Brozynska M., Furtado A., Waters D.L., Henry R.J. (2015). Relationships of wild and domesticated rices (*Oryza* AA genome species) based upon whole chloroplast genome sequences. Sci. Rep..

[31] Murray M.G., Thompson W.F. (1980). Rapid isolation of high molecular weight plant DNA. Nucleic Acids Res..

[32] Andrews S. FASTQC. A Quality Control Tool for High throughput Sequence Data. https://www.bioinformatics.babraham.ac.uk/projects/fastqc/.

[33] Zerbino D.R., Birney E. (2008). Velvet: Algorithms for de novo short read assembly using de Bruijn graphs. Genome Res..

[34] Kearse M., Moir R., Wilson A., Stones-Havas S., Cheung M., Sturrock S., Buxton S., Cooper A., Markowitz S., Duran C. (2012). Geneious basic: An integrated and extendable desktop software platform for the organization and analysis of sequence data. Bioinformatics.

[35] Tillich M., Lehwark P., Pellizzer T., Ulbricht-Jones E.S., Fischer A., Bock R., Greiner S. (2017). GeSeq-versatile and accurate annotation of organelle genomes. Nucleic Acids Res..

[36] Shi L., Chen H., Jiang M., Wang L., Wu X., Huang L., Liu C. (2019). CPGAVAS2, an integrated plastome sequence annotator and analyzer. Nucleic Acids Res..

[37] Lowe T.M., Chan P.P. (2016). tRNAscan-SE On-line: Integrating search and context for analysis of transfer RNA genes. Nucleic Acids Res..

[38] Laslett D., Canback B. (2004). ARAGORN, a program to detect tRNA genes and tmRNA genes in nucleotide sequences. Nucleic Acids Res..

[39] Cheong W.-H., Tan Y.-C., Yap S.-J., Ng K.-P. (2015). Clico FS: An interactive web-based service of Circos. Bioinformatics.

[40] Li H., Durbin R. (2009). Fast and accurate short read alignment with burrows-wheeler transform. Bioinformatics.

[41] Milne I., Bayer M., Cardle L., Shaw P., Stephen G., Wright F., Marshall D. (2009). Tablet-next generation sequence assembly visualization. Bioinformatics.

[42] Zhang Z., Zhao W., Xiao J., Bao Y., He S., Zhang G., Li Y., Zhao G., Chen R., Gao Y. (2020). Database resources of the national genomics data center in 2020. Nucleic Acids Res..

[43] Katoh K., Kuma K.I., Toh H., Miyata T. (2005). MAFFT version 5: Improvement in accuracy of multiple sequence alignment. Nucleic Acids Res..

[44] Rozas J., Ferrer-Mata A., Sanchez-DelBarrio J.C., Guirao-Rico S., Librado P., Ramos-Onsins S.E., Sanchez-Gracia A. (2017). DnaSP 6: DNA sequence polymorphism analysis of large data sets. Mol. Biol. Evol..

[45] Amiryousefi A., Hyvönen J., Poczai P. (2018). IRscope: An online program to visualize the junction sites of chloroplast genomes. Bioinformatics.

[46] Darzentas N. (2010). Circoletto: Visualizing sequence similarity with Circos. Bioinformatics.

[47] Mower J.P. (2009). The PREP suite: Predictive RNA editors for plant mitochondrial genes, chloroplast genes and user-defined alignments. Nucleic Acids Res..

[48] Beier S., Thiel T., Münch T., Scholz U., Mascher M. (2017). MISA-web: A web server for microsatellite prediction. Bioinformatics.

[49] Kurtz S., Choudhuri J.V., Ohlebusch E., Schleiermacher C., Stoye J., Giegerich R. (2002). REPuter: The manifold applications of repeat analysis on a genomic scale. Nucleic Acids Res..

[50] Benson G. (1999). Tandem repeats finder: A program to analyze DNA sequences. Nucleic Acids Res..

[51] Nguyen L.-T., Schmidt H.A., von Haeseler A., Minh B.Q. (2015). IQ-TREE: A fast and effective stochastic algorithm for estimating maximum-likelihood phylogenies. Mol. Biol. Evol..

[52] Kalyaanamoorthy S., Minh B.Q., Wong T.K.F., von Haeseler A., Jermiin L.S. (2017). ModelFinder: Fast model selection for accurate phylogenetic estimates. Nat. Methods.

[53] Hoang D.T., Chernomor O., von Haeseler A., Minh B.Q., Vinh L.S. (2018). UFBoot2: Improving the ultrafast bootstrap approximation. Mol. Biol. Evol..

[54] Dereeper A., Guignon V., Blanc G., Audic S., Buffet S., Chevenet F., Dufayard J.-F., Guindon S., Lefort V., Lescot M. (2008). Phylogeny.fr: Robust phylogenetic analysis for the non-specialist. Nucleic Acids Res..

[55] Lemoine F., Correia D., Lefort V., Doppelt-Azeroual O., Mareuil F., Cohen-Boulakia S., Gascuel O. (2019). NGPhylogeny.fr: New generation phylogenetic services for non-specialists. Nucleic Acids Res..

[56] Lanfear R., Calcott B., Ho S.Y.W., Guindon S. (2012). PartitionFinder: Combined selection of partitioning schemes and substitution models for phylogenetic analyses. Mol. Biol. Evol..

[57] Huelsenbeck J.P., Ronquist F. (2001). MRBAYES: Bayesian inference of phylogenetic trees. Bioinformatics.

[58] Darriba D., Taboada G.L., Doallo R., Posada D. (2012). jModelTest 2: More models, new heuristics and parallel computing. Nat. Methods.

[59] Qian J., Song J., Gao H., Zhu Y., Xu J., Pang X., Yao H., Sun C., Li X., Li C. (2013). The complete chloroplast genome sequence of the medicinal plant *Salvia miltiorrhiza*. PLoS ONE.

[60] Abdullah, Mehmood F., Shahzadi I., Ali Z., Islam M., Naeem M., Mirza B., Lockhart P., Ahmed I., Waheed M.T. (2020). Correlations among oligonucleotide repeats, nucleotide substitutions and insertion-deletion mutations in chloroplast genomes of plant family *Malvaceae*. J. Syst. Evol..

[61] Shahzadi I., Abdullah, Mehmood F., Ali Z., Ahmed I., Mirza B. (2020). Chloroplast genome sequences of *Artemisia maritima* and *Artemisia absinthium*: Comparative analyses, mutational hotspots in genus *Artemisia* and phylogeny in family *Asteraceae*. Genomics.

[62] Song Y., Chen Y., Lv J., Xu J., Zhu S., Li M. (2019). Comparative chloroplast genomes of *Sorghum* species: Sequence divergence and phylogenetic relationships. Biomed. Res. Int..

[63] Sun J., Chen M., Yujiang, Zhao D., Tao J. (2018). Characterization of the complete chloroplast genomes of sequences of two diploid species: *Paeonia lactiflora* ‘Da Fugui’ and *Paeonia ostii* ‘Fengdan’ in the *Paeoniaceae* Family. J. Hortic..

[64] Ahmed I., Biggs P.J., Matthews P.J., Collins L.J., Hendy M.D., Lockhart P.J. (2012). Mutational dynamics of aroid chloroplast genomes. Genome Biol. Evol..

[65] Shaw J., Lickey E.B., Schilling E.E., Small R.L. (2007). Comparison of whole chloroplast genome sequences to choose noncoding regions for phylogenetic studies in angiosperms: The tortoise and the hare III. Am. J. Bot..

[66] Hollingsworth P.M., Graham S.W., Little D.P. (2011). Choosing and using a plant DNA barcode. PLoS ONE.

[67] Worberg A., Quandt D., Barniske A.M., Löhne C., Hilu K.W., Borsch T. (2007). Phylogeny of basal eudicots: Insights from non-coding and rapidly evolving DNA. Org. Divers. Evol..

[68] Powell W., Morgante M., McDevitt R., Vendramin G.G., Rafalski J.A. (1995). Polymorphic simple sequence repeats regions in chloroplast genomes: Applications to the population genetics of pines. Proc. Natl. Acad. Sci. USA.

[69] Xue J., Wang S., Zhou S.L. (2012). Polymorphic chloroplast microsatellite loci in *Nelumbo* (*Nelumbonaceae*). Am. J. Bot..

[70] Zhang Y., Du L., Liu A., Chen J., Wu L., Hu W., Zhang W., Kim K., Lee S.-C., Yang T.-J. (2016). The Complete chloroplast genome sequences of five *Epimedium* species: Lights into phylogenetic and taxonomic analyses. Front. Plant. Sci..

[71] Wang R.J., Cheng C.L., Chang C.C., Wu C.L., Su T.M., Chaw S.M. (2008). Dynamics and evolution of the inverted repeat-large single copy junctions in the chloroplast genomes of monocots. BMC Evol. Biol..

[72] Palmer J.D., Jansen R.K., Michaels H.J., Chase M.W., Manhart J.R. (1988). Chloroplast DNA variation and plant phylogeny. Ann. Mo. Bot. Gard..

[73] Liu H., He J., Ding C., Lyu R., Pei L., Cheng J., Xie L. (2018). Comparative analysis of complete chloroplast genomes of *Anemoclema*, *Anemone*, *Pulsatilla*, and *Hepatica* revealing structural variations among genera in tribe *Anemoneae* (*Ranunculaceae*). Front. Plant. Sci..

[74] Bundschuh R., Altmüller J., Becker C., Nürnberg P., Gott J.M. (2011). Complete characterization of the edited transcriptome of the mitochondrion of *Physarum polycephalum* using deep sequencing of RNA. Nucleic Acids Res..

[75] Zeng W.H., Liao S.C., Chang C.C. (2007). Identification of RNA editing sites in chloroplast transcripts of *Phalaenopsis aphrodite* and comparative analysis with those of other seed plants. Plant. Cell Physiol..

[76] Kool A., Oxelman B., Thulin M. Phylogeny of *Withania* (*Solanaceae*). Proceedings of the Abstracts XVII International Botanical Congress.

[77] Jamil I., Qamarunnisa S., Azhar A., Shinwari Z.K., Ali S.I., Qaiser M., Jamil I., Al E.T. (2014). Subfamilial relationships within *Solanaceae* as inferred from *atp*B-*rbc*L intergenic spacer. Pak. J. Bot..

[78] Jansen R.K., Cai Z., Raubeson L.A., Daniell H., de Pamphilis C.W., Leebens-Mack J., Muller K.F., Guisinger-Bellian M., Haberle R.C., Hansen A.K. (2007). Analysis of 81 genes from 64 plastomes resolves relationships in angiosperms and identifies genome-scale evolutionary patterns. Proc. Natl. Acad. Sci. USA.

[79] Olmstead R.G., Sweere J.A., Spangler R.E., Bohs L., Palmer J.D., Nee M., Symon D.E., Lester R.N., Jessop J.P. (1999). Phylogeny and provisional classification of the Solanaceae based on chloroplast DNA. Solanaceae IV: Advances in Biology and Utilization.

[80] D’Arcy W.G., Hawkes J.G., Lester R.N., Nee M., Estrada N. (1991). The *Solanaceae* since 1976, with a review of its biogeography. Solanaceae III: Taxonomy, Chemistry, Evolution.

[81] Symon D.E., Hawkes J.G., Lester R.N., Nee M., Estrada N. (1991). Gondwanan elements of the *Solanaceae*. Solanaceae III: Taxonomy, Chemistry, Evolution.

[82] Deanna R., Smith S.D., Särkinen T., Chiarini F. (2018). Patterns of chromosomal evolution in the florally diverse Andean clade *Iochrominae* (*Solanaceae*). Perspect. Plant. Ecol. Evol. Syst..

[83] Hepper F.N., Hakes J.G., Lester R.N., Nee M., Estrada N. (1991). Old world *Withania* (*Solanaceae*): A taxonomic review and key to the species. Solanaceae III: Taxonomy, Chemistry, Evolution.

[84] Hunziker A.T. (2001). Genera Solanacearum: The Genera of Solanaceae Illustrated, Arranged According to a New System.

[85] Choi K.S., Chung M.G., Park S. (2016). The complete chloroplast genome sequences of three veroniceae species (*Plantaginaceae*): Comparative analysis and highly divergent regions. Front. Plant. Sci..

[86] Abdullah, Waseem S., Mirza B., Ahmed I., Waheed M.T. (2020). Comparative analyses of chloroplast genome in *Theobroma cacao* and *Theobroma grandiflorum*. Biologia.

[87] Henriquez C.L., Abdullah, Ahmed I.A., Carlsen M.M., Zuluaga A., Croat T.B., Mckain M.R. (2020). Molecular evolution of chloroplast genomes in *Monsteroideae* (*Araceae*). Planta.

[88] Ahmed I., Matthews P.J., Biggs P.J., Naeem M., Mclenachan P.A., Lockhart P.J. (2013). Identification of chloroplast genome loci suitable for high-resolution phylogeographic studies of *Colocasia esculenta* (L.) Schott (*Araceae*) and closely related taxa. Mol. Ecol. Resour..

[89] Dong W., Liu J., Yu J., Wang L., Zhou S. (2012). Highly variable chloroplast markers for evaluating plant phylogeny at low taxonomic levels and for DNA barcoding. PLoS ONE.

[90] Nguyen V.B., Park H.-S., Lee S.-C., Lee J., Park J.Y., Yang T.-J. (2017). Authentication markers for five major *Panax* species developed via comparative analysis of complete chloroplast genome sequences. J. Agric. Food Chem..

[91] Kimura M. (1979). Model of effectively neutral mutations in which selective constraint is incorporated. Proc. Natl. Acad. Sci. USA.

[92] Lawrie D.S., Messer P.W., Hershberg R., Petrov D.A. (2013). Strong purifying selection at synonymous sites in *D. melanogaster*. PLoS Genet..

[93] Poczai P., Hyvönen J. (2017). The complete chloroplast genome sequence of the CAM epiphyte Spanish moss (*Tillandsia usneoides*, *Bromeliaceae*) and its comparative analysis. PLoS ONE.

[94] Mehmood F., Abdullah, Ubaid Z., Shahzadi I., Ahmed I., Waheed M.T., Poczai P., Mirza B. (2020). Plastid genomics of *Nicotiana* (*Solanaceae*): Insights into molecular evolution, positive selection and the origin of the maternal genome of Aztec tobacco (*Nicotiana rustica*). BioRxiv.

[95] Abdullah, Mehmood F., Shahzadi I., Waseem S., Mirza B., Ahmed I., Waheed M.T. (2020). Chloroplast genome of *Hibiscus rosa-sinensis* (*Malvaceae*): Comparative analyses and identification of mutational hotspots. Genomics.

[96] Spooner D.M. (2009). DNA barcoding will frequently fail in complicated groups: An example in wild potatotes. Am. J. Bot..

[97] Miller J.T., Spooner D.M. (1999). Collapse of species boundaries in the wild potato *Solanum brevicaule* complex (*Solanaceae*, *S.* sect. *Petota*): Molecular data. Plant. Syst. Evol..

[98] Aubriot X., Knapp S., Syfert M.M., Poczai P., Buerki S. (2018). Shedding new light on the origin and spread of the brinjal eggplant (*Solanum melongena* L.) and its wild relatives. Am. J. Bot..

[99] Kulcheski F.R., Muschner V.C., Lorenz-Lemke A.P., Stehmann J.R., Bonatto S.L., Salzano F.M., Freitas L.B. (2006). Molecular phylogenetic analysis of *Petunia* Juss. (*Solanaceae*). Genetica.

[100] Sullivan J.R. (1985). Systematics of the *Physalis viscosa* complex (*Solanaceae*). Syst. Bot..

